# Mikrobiota a Otyłość

**DOI:** 10.34763/devperiodmed.20172103.203207

**Published:** 2017-10-28

**Authors:** Alicja Karney

**Affiliations:** 1 Oddział Hospitalizacji Jednego Dnia, Instytut Matki i Dziecka, Warszawa, Polska

**Keywords:** mikrobiota, otyłość, microbiota, obesity

## Abstract

Nadwaga i otyłość z ich konsekwencjami pod postacią cukrzycy typu 2 i chorób układu krążenia, stanowią duży problem zdrowia publicznego. Według ostatnich doniesień ważną rolę w „epidemii” otyłości może odgrywać mikrobiota jelitowa. Z uwagi na to, że mikrobiota jelit może wpływać na masę ciała, wrażliwość na insulinę czy metabolizm glukozy i lipidów pozwala wysunąć hipotezę, że zmiany w obrębie mikrobioty mogą mieć znaczenie w patogenezie otyłości i zespołu metabolicznego.

## Abstract (en)

Overweight and obesity can have serious consequences that are a major public health problem, such as e.g. type 2 diabetes and cardiovascular disease. According to recent reports, gut microbiota may play an important role in the “epidemic” of obesity. The fact that intestinal microbiota may influence body weight, insulin sensitivity or glucose and lipid metabolism has led to the hypothesis that these changes may contribute to the pathogenesis of obesity and metabolic syndrome.

Nadwaga i otyłość oraz ich konsekwencje pod postacią m.in. chorób układu krążenia, zaburzeń lipidowych czy cukrzycy typu II, stanowią olbrzymi problem zdrowia publicznego od kilku lat. Rozwój otyłości ma związek z wieloma czynnikami, zarówno genetycznymi, jak i środowiskowymi. Od kilku lat podnosi się rolę jelita i organizmów go zasiedlających w patomechanizmie otyłości.

Przewód pokarmowy człowieka jest skolonizowany przez kompleks 10 mld drobnoustrojów nazywany kiedyś mikroflorą jelitową. Pojęcie mikrobiomu zostało wprowadzone w 2001 r. przez Joshua Lederberg’a i określało całość ekologicznego środowiska złożonego z drobnoustrojów komensalicznych, symbiotycznych i chorobotwórczych. Obecnie termin ten określa zespół wszystkich genów mikroorganizmów żyjących w i na ciele człowieka, natomiast zespół tych mikrobów określa się pojęciem mikrobioty jelitowej [1]. W 2007 r. został zainicjowany projekt − HMP (Human Microbiome Project), którego głównym celem jest badanie i opisanie mikrobiomu człowieka w stopniu, który umożliwi badanie jego zmian w odniesieniu do populacji, genotypu, stanu zdrowia, wieku, rodzaju diety, stosowanych leków i środowiska oraz jego wpływu na różne choroby [2, 3].

Do badania mikrobioty człowieka wykorzystuje się analizę 16S rRNA oraz bada się złożoność próbek na drodze sekwencjonowania materiału genetycznego uzyskanego bezpośrednio ze środowiska. To podejście jest nazywane „metagenomiką”. Termin metagenomika został zaproponowany przez prof. J.Handelsma’a w roku 1998 [4].

Ogromną rolę w badaniach, oprócz metagenomiki, odgrywają również badania mRNA (meta-transkryptomika), białek (metaproteomika) i sieci metabolicznych (metainteraktomika), z uwagi na to, że sama metagenomika nie zapewnia bezpośrednich informacji o tym, które geny są w danych warunkach funkcjonalne [4].

Metagenomowe analizy ludzkiego mikrobiomu wykazały, że w jelicie znajduje się 3,3 miliona unikatowych genów, czyli 150 razy więcej niż w naszym własnym genomie, a różnorodność bakterii jelitowych jest szacowana na ponad 1000 gatunków [5, 6].

Badania, dzięki którym, możliwe jest poznanie wpływu mikrobioty na organizm ludzki są możliwe dzięki badaniom na myszach germ-free (myszy wolne od mikroflory, hodowane w warunkach sterylnych) oraz analizie genu 16SrRNA u wielu mikroorganizmów. Gen ten, wielkości 1,5 kb, zawiera w swojej strukturze silnie konserwowane sekwencje, utrwalane w toku ewolucji. Służy on do klasyfikacji mikroorganizmów i pozwala udokumentować historię ich ewolucji oraz tworzenie drzew filogenetycznych [7, 8]. Grupowania i segregowania filotypów dokonano na podstawie tzw. „podobieństwa sekwencji” (5ID) genów 16SrRNA. Badania nad genem 16S rRNA, wykonane w ciągu ostatnich dziesięciu lat, stały się punktem zwrotnym w poznaniu różnorodności bakteryjnej mikroflory znajdującej się w ludzkim przewodzie pokarmowym [9].

Kompleksowe badania pozwoliły sklasyfikować 4 typy mikroorganizmów jelitowych, które stanowią 94-98% wszystkich izolowanych drobnoustrojów, są to: Firmicutes (64%), Bacteroides(23%), Proteobacteria(8%), Actinobacteria (3%). Dominują Bacteroides i Firmicutes. Bakterie te są obecne w całym przewodzie pokarmowym, ale największa ich liczba jest w jelicie grubym − między 10 do 10 a 10 do 12 komórek/gram treści pokarmowej [10].

Do niedawna uważano, że noworodek, a szczególnie jego jelito jest jałowe. W 2014 r. pojawiły się doniesienia o obecności bakterii w łożysku i płynie owodniowym w okresie prenatalnym. Uważa się, że pochodzą one z przewodu pokarmowego matki [11, 12, 13]. Według doniesień na rozwój mikrobioty noworodka mają wpływ czynniki zewnętrzne, takie, jak rodzaj porodu (poród silami natury, ciecie cesarskie), rodzaj karmienia niemowlęcia (naturalny, mleko modyfikowane), spożycie antybiotyków przez matkę w czasie ciąży oraz u noworodka [14]. Kształtowanie się mikrobioty dziecka trwa do 2-3 roku życia. W pierwszej dobie życia w jelitach noworodka dominują E. coli i Enterokoki, w 2 dobie życia u niemowląt karmionych naturalnie pojawiają się Bifidobakterie, Lactobacillus, Bacteroides i Clostridium. U dzieci karmionych mlekiem modyfikowanym bakterie z rodzaju Bifidobacterium i Lactobacillus pojawiają się dopiero około 30 dnia życia dziecka, a nawet później [14].

Mleko kobiece można zaliczyć do naturalnych synbiotykow, bowiem zawiera naturalne oligosacharydy − HMO (Human milk oligosacharydes) i bakterie probiotyczne, wpływające na skład mikroflory jelitowej dziecka. U noworodków urodzonych silami natury w 3 dobie życia zmniejsza się liczebność bakterii z rodzaju Clostridium na korzyść Bifidobacterium [15, 16].

Noworodek urodzony przez cięcie cesarskie jest pozbawiony kontaktu z mikrobiotą jelitową matki, co ma wpływ na skład jego mikrobioty jelitowej. Po porodzie w jelicie noworodków częściej hoduje się Clostridium dificile oraz bakterie ze środowiska szpitalnego [16]. Zmieniony skład mikrobioty u niemowląt z cięć cesarskich może mieć charakter długofalowy, wg Salminena nawet do 7 rż. [17]. Wiadomo również, że poród dziecka przez cięcie cesarskie zwiększa ryzyko otyłości u dzieci o ok. 46% [18].

Ważnym czynnikiem wpływającym na skład mikrobioty niemowlęcia jest antybiotykoterapia matki w czasie ciąży oraz w pierwszych tygodniach życia dziecka. Przyjmowanie antybiotyków w czasie 2 i 3 trymestru ciąży przez kobiety zwiększa ryzyko rozwoju otyłości o 84% u potomstwa w porównaniu z grupą dzieci, których matki nie przyjmowały antybiotyków. Według badań, antybiotykoterapia może mieć wpływ na ryzyko wzrostu masy ciała u dzieci w wieku 2 lat, zwłaszcza dotyczy to antybiotyków makrolidowych podawanych w pierwszych 6 miesiącach życia i częściej niż 1 raz [14].

Badania kohortowe KOALA z 2002r. (n=2834) wykazały, że skład mikrobioty jelitowej we wczesnym dzieciństwie wpływa na masę ciała dzieci w późniejszych latach, a dokładniej − kolonizacja Bacteroides fragilis u dzieci w 1 miesiącu życia wiąże się z wyższym BMI dzieci w wieku 10 lat. Dotyczyło to dzieci, których matki miały małą ilość błonnika w diecie w okresie ciąży [19]. Około 2-3 rż. mikrobiota jelitowa stabilizuje się i staje się podobna do flory osoby dorosłej, z dominacją Bacteroitedes.

Jednym z najistotniejszych czynników wpływających na skład mikroflory jelitowej jest dieta. Wiele przeprowadzonych badań wykazuje, że zmiana diety z nisko- na wysokotłuszczową powodowała znaczące różnice ilościowe w składzie mikrobioty jelitowej. Obserwowano spadek liczebności bakterii należących do typu Bacteroidetes przy jednoczesnym, znaczącym wzroście liczebności Firmicutes i Proteobacteria. Zmiany te były niezależne od występowania, bądź braku objawów otyłości u badanych osób [20]. Podobne wyniki uzyskali naukowcy porównujący ( orę bakteryjną dzieci karmionych wysokoenergetyczną dietą zachodnią oraz dzieci z regionów afrykańskich [21].

W ostatnich latach mikrobiota jelitowa została zidentyfikowana jako determinanta rozwoju otyłości, zarówno w badaniach na zwierzętach, jak i na ludziach. Badania Ley’a i wsp., opublikowane w 2005 r. pokazały po raz pierwszy różnice w mikrobiocie jelit myszy szczupłych i otyłych [22]. Mikrobiota myszy otyłych zawierała znacząco mniej bakterii z rodzaju Bacteroides a wiecej Firmicutes w porównaniu z ich szczupłymi bliźniakami. Ponadto, kolonizacja myszy germ-free mikrobiotą pobraną od otyłych myszy, prowadziłado znacznie większego odkładania się tłuszczu w porównaniu z myszami szczupłymi.

Prowadzono również badania przeniesienia ludzkiej mikrobioty jelitowej do jelit myszy germ-free. Skolonizowanie ich ludzką mikrobiotą powodowało, że przekazywały cechę potomstwu i dodatkowo zwiększały różnorodność mikrobioty. Porównania sekwencji 16S rRNA bakterii z próbek kału ludzi dorosłych o różnym stopniu pokrewieństwa, pokazały m.in. wyraźnie wyższe podobieństwo mikroflory jelit pomiędzy monozygotycznymi bliźniakami niż w przypadku niespokrewnionych osób żyjących w takich samych warunkach środowiskowych np. małżeństw [23]. Natomiast porównania zestawów microbiota pobranych od mono- i dizygotycznych bliźniąt jeszcze wyraźniej wskazały na duże znaczenie genotypu gospodarza [24].

Kolejne badania polegały na badaniu składu mikrobioty jelit myszy po wprowadzeniu diety wysokotłuszczowej. Okazało się, że dieta taka powoduje zmiany składu mikrobioty jelita już po upływie jednego dnia. Badania wykazały również możliwość wywołania otyłości u zwierząt na skutek przeniesienia mikrobioty ludzi otyłych do jelit zdrowych zwierząt [25].

Turnbaugh i wsp. udowodnili, że skład mikrobioty jelitowej wpływa na masę ciała. Przeprowadzili oni transfer mikroorganizmów z jelit homozygotycznych myszy otyłych ob/ob (myszy z genetycznie uwarunkowanym brakiem leptyny wynikającym z mutacji typu nonsens w 105 kodonie genu ob.) i myszy o prawidłowej masie ciała do jelit myszy germ free (wolne od wszystkich wykrywalnych mikroorganizmów i pasożytów) o prawidłowej masie ciała. Po dwóch tygodniach zaobserwowano, że myszy, którym przeszczepiono mikroflorę od myszy otyłych, pozyskiwały więcej kalorii z pożywienia i wykazywały szybsze odkładanie tkanki tłuszczowej. Myszy otyłe w porównaniu z myszami szczupłymi miały o 50% mniejszą zawartość Bacteroides i proporcjonalny wzrost Firmicutes, zaobserwowano również wzrost genów związanych z wykorzystaniem energii z pożywienia, co może być przyczyną rozwoju otyłości [26, 27].

W innym eksperymencie wykazano, że w przeciwieństwie do myszy posiadających mikroflorę jelit, zwierzęta GF nie mają tendencji do tycia, pomimo spożywanej wysokotłuszczowej i wysokocukrowej diety [28]. Kolejne badania wcześniej cytowanego Ley’a i wsp. wykazały, że dieta bogata w tłuszcze zwiększa endotoksemię, redukuje liczbę bakterii Gram-ujemnych (Bacteroides spp.), Gram-dodatnich (Eubacterium recitale), indukuje rozwój otyłości, powstawanie insulinooporności i cukrzycy. U osób będących na niskokalorycznej diecie, wraz z utratą wagi, wzrasta w ich jelitach liczba bakterii z typu Bacteroides. Obserwacje te jednak nie wykazały jednoznacznie: czy zmiana w zawartości Bacteroides jest przyczyną tycia, czy był to efekt spożywania określonych składników pokarmowych, które selektywnie wspomagały wzrost jednych grup bakterii, nie wpływając lub hamując wzrost innych [29].

Mikrobiota jelitowa wpływa na homeostazę energetyczną organizmu gospodarza, co jest nazywane „hipotezą magazynowania” (the storage hypothesis) [30]. Jest kilka mechanizmów, które wyjaśniają interakcje między mikrobiotą a metabolizmem gospodarza mogących przyczynić się do rozwoju otyłości. Dzięki syntezie i wydzielaniu wielu związków chemicznych, mikrobiota może wpływać na podwojenie gęstości naczyń włosowatych w nabłonku jelita cienkiego, co skutkuje zwiększonym wchłanianiem monosacharydów w tym odcinku przewodu pokarmowego [31]. Dzięki symbiozie człowieka i bakterii jelitowych możliwe jest pozyskiwanie energii ze związków, które nie są rozkładane przez enzymy trawienne, a które w wyniku fermentacji przeprowadzanej przez mikrobiotę, dostają się do krwiobiegu. Związkami tymi są m.in. krótkołańcuchowe kwasy tłuszczowe (SCFAs − short chain fatty acids), takie, jak: octan, propionian i maślan. Propionian może być wykorzystywany w procesie syntezy glukozy i lipidów [32]. Krótkołańcuchowe kwasy tłuszczowe stymulują wydzielanie peptydu YY (PYY − peptide YY), który spowalniając motorykę jelit zwalniają przechodzenie treści pokarmowej w jelitach, a przez to zwiększają wchłanianie składników odżywczych [32]. Uważa się, że peptyd YY, będący hormonem, może mieć wpływ na rozwój otyłości [32]. Z kolei maślan ma wpływ na regulację homeostazy energetycznej organizmu poprzez stymulację wytwarzania leptyny w adipocytach i indukcję wydzielania GLP-1 przez komórki L jelita, a także nasilenie procesu termogenezy, wzrost utleniania kwasów tłuszczowych oraz aktywności mitochondriów w obrębie mięśni i brunatnej tkanki tłuszczowej [33]. U otyłych myszy karmionych wysokotłuszczową dietą wzbogaconą o maślan zaobserwowano zahamowanie, a nawet cofnięcie się insulinooporności [34]. W innym badaniu zaobserwowano zaś, że spożywanie diety o niskiej zawartości węglowodanów skutkuje obniżonym stężeniem kwasu masłowego w próbkach kału oraz zmniejszeniem liczby bakterii, które go wytwarzają. Na podstawie tych informacji można wysunąć hipotezę, że maślan korzystnie wpływa na metabolizm w stanach patologicznych, natomiast nie odgrywa większej roli w warunkach prawidłowych [35]. Uważa się również, że mikrobiota jelit może sprzyjać magazynowaniu tłuszczu poprzez blokowanie ekspresji czynnika tkankowego indukowanego głodzeniem (FIAF − Fasting-induced adipocyte factor). FIAF, znany także pod nazwą białka podobnego do angiopoetyny 4, hamuje działanie lipazy lipoproteinowej (LPL − lipoprotein lipase) – enzymu odpowiedzialnego za magazynowanie energii w postaci tłuszczu. Ponadto FIAF ułatwia uwalnianie kwasów tłuszczowych ze związanych z lipoproteinami trójglicerydów, a w związku z tym obniża ekspresję FIAF i prowadzi do zwiększenia aktywności LPL w komórkach tłuszczowych oraz nasila proces magazynowania energii w postaci tłuszczu [36]. Mikrobiota jelitowa może również wpływać na metabolizm lipidów gospodarza, hamując aktywność kinazy białkowej aktywowanej przez AMP (AMPK − adenosine monophosphate activated protein kinase). AMPK kontroluje status energetyczny na poziomie komórkowym [36]. Badania wykazały, że dzięki dużej aktywności ufosforylowanej postaci AMPK w wątrobie i mięśniach szkieletowych myszy, a więc wysokiej wydajności utleniania kwasów tłuszczowych w obu tych organach, myszy germ-free, mimo karmienia dietą wysokotłuszczową i wysokowęglowodanową, bronią się przed otyłością [36]. Kwasy żółciowe produkowane przez wątrobę, poprzez aktywację pewnych receptorów, mają również duży wpływ na metabolizm lipidów i glukozy. W badaniach obserwowano, że u myszy germ-free są obecne zmiany w proporcji kwasów żółciowych w porównaniu z osobnikami hodowanymi w „normalnych”, niesterylnych warunkach [37]. Pierwszym zidentyfikowanym receptorem jądrowym był aktywowany przez kwasy żółciowe FXR (farnesoid X receptor). We krwi myszy pozbawionych tego receptora (FXR-/-) obserwuje się podwyższone stężenia trójglicerydów i glukozy [37]. Innym receptorem aktywowanym przez kwasy żółciowe jest TGR5. Jest on receptorem błonowym, wykrywanym głównie w brunatnej tkance tłuszczowej i jelicie cienkim. Przekazywanie sygnału poprzez TGR5 powoduje zwiększenie poziomu cAMP, a to z kolei prowadzi do wzmożonego zużycia energii w obrębie brunatnej tkanki tłuszczowej, a więc może zapobiegać powstawaniu insulinooporności i otyłości [38].

Wykazano, że popularne diety mające na celu zmniejszenie masy ciała, oparte na spożywaniu przede wszystkim białek i małej ilości węglowodanów, mogą powodować zmianę składu populacji bakterii w jelicie grubym człowieka i ich mikrobiologicznej aktywności, a co za tym idzie, ich wpływu na zdrowie gospodarza. Wprowadzając określone diety i pobierając próbki kału od badanych osób do analizy 16S rRNA przy pomocy ( uorescencyjnej hybrydyzacji in-situ (FISH), wykazano, że redukcja ilości węglowodanów w spożywanych posiłkach prowadzi do spadku zawartości w jelitach bakterii produkujących maślan: Roseburia spp., Eubacterium recitale oraz Bifidobacterium spp., ale nie stymuluje zmian liczby Bacteroides spp., zaś spożywanie większej ilości węglowodanów skutkuje zwiększeniem ogólnej liczby komórek bakterii [39, 40].

Potwierdza to hipotezę, że bakteryjna mikroflora nie tylko umożliwia wydajniejsze wykorzystywanie węglowodanów zawartych w pożywieniu, ale też ma zdolność do modulowania przetwarzania pokarmu i magazynowania tłuszczu przez gospodarza [27, 28, 36].

## Podsumowanie

Mikrobiota przewodu pokarmowego, nazywana przez badaczy „nowym organem w obrębie ludzkiego organizmu”, ma znaczący wpływ na funkcjonowanie organizmu od okresu prenatalnego. Nowoczesne badania umożliwiają lepsze zrozumienie funkcji mikrobioty jelitowej, w szczególności w świetle narastającej w świecie otyłości i związanych z nią powikłań.
